# The Impact of Non-Pathogenic Bacteria on the Spread of Virulence and Resistance Genes

**DOI:** 10.3390/ijms24031967

**Published:** 2023-01-19

**Authors:** Francisco Dionisio, Célia P. F. Domingues, João S. Rebelo, Francisca Monteiro, Teresa Nogueira

**Affiliations:** 1 cE3c—Centre for Ecology, Evolution and Environmental Changes & CHANGE, Global Change and Sustainability Institute, Faculdade de Ciências, Universidade de Lisboa, 1749-016 Lisboa, Portugal; 2 INIAV—National Institute for Agrarian and Veterinary Research, 2780-157 Oeiras, Portugal

**Keywords:** antibiotic resistance, virulence, microbiome, metagenomics, human gut, antibiotic consumption

## Abstract

This review discusses the fate of antimicrobial resistance and virulence genes frequently present among microbiomes. A central concept in epidemiology is the mean number of hosts colonized by one infected host in a population of susceptible hosts: *R*_0_. It characterizes the disease’s epidemic potential because the pathogen continues its propagation through susceptible hosts if it is above one. *R*_0_ is proportional to the average duration of infections, but non-pathogenic microorganisms do not cause host death, and hosts do not need to be rid of them. Therefore, commensal bacteria may colonize hosts for prolonged periods, including those harboring drug resistance or even a few virulence genes. Thus, their *R*_0_ is likely to be (much) greater than one, with peculiar consequences for the spread of virulence and resistance genes. For example, computer models that simulate the spread of these genes have shown that their diversities should correlate positively throughout microbiomes. Bioinformatics analysis with real data corroborates this expectation. Those simulations also anticipate that, contrary to the common wisdom, human’s microbiomes with a higher diversity of both gene types are the ones that took antibiotics longer ago rather than recently. Here, we discuss the mechanisms and robustness behind these predictions and other public health consequences.

## 1. Introduction

“*What is essential is invisible to the eye*”.

Antoine de Saint-Exupéry.

“The Little Prince” (1943) [1].

Antibiotic-resistant bacteria caused 1.27 million human deaths worldwide in 2019 [2]. This estimation indicates, once more, that antimicrobial resistance is a severe world health problem [3,4]. Bacterial pathogens are, by definition, the etiological agents of bacterial diseases. However, one must consider commensal bacteria in the list of public health concerns because they are also reservoirs of genes encoding adaptive traits (non-housekeeping genes), e.g., virulence, heavy-metal resistance, and antibiotic-resistance genes that can transfer to pathogenic bacteria [5,6,7,8,9,10,11]. For example, a recent work involving bacteria of the *Neisseria* genus has shown that the phenotypic resistance profile of four commensal *Neisseria* species was higher than that of four pathogenic *Neisseria gonorrhoeae* strains [12].

Gene transfer occurs through three major mechanisms. Bacteria can directly uptake DNA from the environment or with the connivance of vectors such as conjugative plasmids, conjugative integrative elements, or bacteriophages [13,14]. For example, in a study of the donor ability of a naturally isolated conjugative plasmid conferring resistance to six antibiotics between 14 enterobacteria, the best plasmid donor was a commensal *Escherichia coli* [7].

These DNA transfer events may occur between different bacteria within microbiomes. Human microbiomes–the collection of all microorganisms (and their genomes) living in human tissues–are complex [15], comprising more than 10^13^ bacterial cells belonging to hundreds of species [16]. In the human body, microorganisms are especially numerous and diverse in the skin, mucosa, and gastrointestinal tract, possibly comprising both pathogenic and non-pathogenic bacteria [17]. The metagenome refers to the set of all genes in a microbiome, including chromosomal genes and extra-chromosomal genetic elements, e.g., bacteriophages, transposons, plasmids, and other mobile genetic elements. Several microbiomes can also be very complex, namely that of other animals, soil, plant roots, sewage, etc.

Generally, in human and veterinary medicine, the onset of a symptomatic infectious disease leads to the prescription of antibiotics. However, we argue here that this does not necessarily mean that bacterial pathogenicity is directly associated with antibiotic resistance (see next sections).

Consider the following arguments:

(i) People that have not used antibiotics for a long time have a lower diversity of drug-resistance genes in their microbiomes;
(ii) The diversity of resistance genes in a person’s microbiome increases when that person takes an antibiotic;
(iii) Antibiotic overuse increases the diversity of resistance genes in human microbiomes;
(iv) The co-location of virulence and resistance genes in bacterial genomes explains the positive correlation between the resistance and virulence genes’ diversities in microbiomes observed over human microbiomes.

Readers that feel some of these arguments are accurate should keep reading this paper. The vast majority of microorganisms in human microbiomes are non-pathogenic, and we will see how they might play critical roles in the spread of virulence and resistance genes. Paraphrasing Antoine de Saint-Exupéry [1], what is essential, is often unnoticed.

## 2. The Fate of Commensal Bacteria

### 2.1. Brief Review of the R_0_, the Basic Reproductive Number

A central concept in the epidemiology of infectious disease transmission is the basic reproductive number: also called the basic reproduction number or basic reproduction ratio. It is the expected number of infected individuals by a single infected individual in a susceptible population. Denoted as *R*_0_, this number is a measure of the pathogen’s fitness but is also a threshold that characterizes the epidemic potential of the disease because: if $R_{0}>1$, the pathogen continues its propagation through susceptible hosts, with the number of infected hosts increasing exponentially; if $R_{0}<1$, the number of infected hosts decreases exponentially, and the pathogen is extinct. The number of infected hosts is stable in the unlikely case where *R_0_* equals one [18,19].

*R_0_* is a dimensionless number defined as $R_{0}=\beta \cdot c\cdot d$, where *β* is the probability of infection if an infected individual contacts a susceptible one, *c* is the number of contacts between infected and susceptible individuals per time unit, and *d* is the duration of the infectious period. Its mathematical formula shows that *R_0_* is proportional to the mean duration of infections *d*. This period decreases when the intrinsic mortality (i.e., mortality not caused by the pathogen) increases, the pathogen-induced mortality increases, and the rate at which the host recovers (e.g., through immunity) increases. When the colonizing agents are pathogens, typically, either the hosts die or manage to clear the pathogen. If the host’s death or recovery is quick, the *R*_0_ of the pathogen is lower than if the pathogen remains a long time in the host. Therefore, there is something peculiar about non-pathogenic microorganisms with public-health consequences.

Non-pathogenic agents do not cause host death, and the hosts do not need to eliminate them. Consequently, commensal or mutualistic bacteria, including those harboring drug resistance or even a few virulence genes, may colonize their hosts for longer, so their *R*_0_ is prone to be larger than one. This conclusion may impact public health because an *R*_0_ larger than one implies that the microorganism may spread through the host population, and as mentioned above, non-pathogenic bacteria may harbor non-housekeeping genes and mobile genetic elements such as virulence and resistance genes.

Even if newly arrived non-pathogenic bacterial cells cannot persist in a microbiome for more than a few days, its mobile genetic elements can have several opportunities to transfer to one of the other established cells in that microbiome [20,21,22], and may thus remain there for a long time. This is possible due to the presence of hundreds of bacterial species in many microbiomes, including the human gut, and because bacteria can receive foreign DNA through three primary mechanisms: (i) transformation, where bacteria directly uptakes DNA from the surroundings [23]; (ii) transduction, where bacteriophages bring DNA from their previous hosts [24]; and (iii) bacterial conjugation, where bacteria receive conjugative plasmids or integrative conjugative elements from neighboring cells [25]. A bacterial cell can uptake DNA from phylogenetically distant bacteria cells, and conjugative plasmids can transfer between cells belonging to different bacterial species [26]. Therefore, both pathogenic and non-pathogenic bacteria can share a gene pool that includes virulence and resistance genes.

Moreover, some bacterial populations containing neither mobile genetic elements nor virulence or resistance genes can become great “amplifiers” of these genes after receiving some plasmids. Some bacterial strains are excellent donors of conjugative plasmids and are able to “amplify” it among them while quickly transmitting the plasmid to other cells in a microbiome [7,27]. These amplifiers are present among strains of *Escherichia coli* and other enterobacterial species [7], among soil bacteria [27], and most probably in the majority of microbiomes.

### 2.2. The Human Network of Physical Contacts

#### 2.2.1. Brief Review of Small-World Networks

People establish many networks involving physical contact with each other through family relationships, friendships, sexual relationships, and many others. Networks have nodes (or vertices) and edges (connections). For example, consider the “handshaking” network in which each node is a person, and two persons are connected in this network if they had at least a handshaking, e.g., last year.

In a typical network established by people, each person connects to a tiny subset of another person included in that network. Therefore, most people have no direct connections (each one of us gave a handshake to just a few people last year).

In some networks involving people, if a given person connects to two others, these two persons are likely connected to each other, but each person can reach most people (through the network’s connections) by a small number of connections. The last sentence sounds somewhat contradictory, but it is not–strikingly, many networks established by people are similar to this–the so-called small-world networks–and we will discuss how that is relevant to understanding microorganisms’ spread.

**Figure 1:**
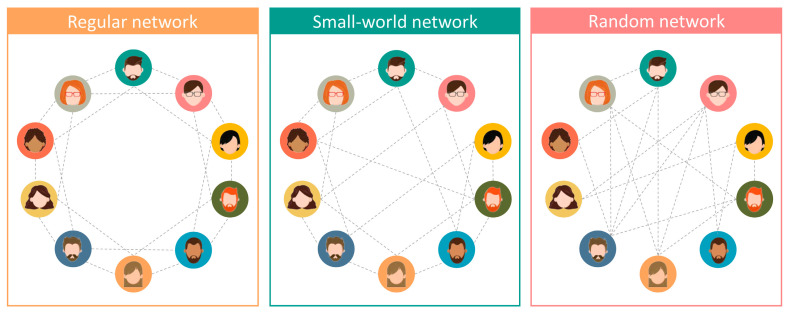
Regular, small-world, and random networks.

We first consider a regular network (Figure 1, left panel) and then change it to make it a small-world network (Figure 1, middle panel). Consider, for example, the network of friendships. For clarification, let us assume that individuals in a population are organized in alphabetic order: A, B, C, …, Y, Z, AA, AB, … and that all individuals have precisely four friends. Frequently, if individual C is a friend of two individuals on his/her right, A and B, and the two individuals on his/her left, D and E, then probably B and D are friends of each other, and A and B or D and E.

Meanwhile, D is a friend of B, C (right), E, and F (left), so B and C are friends to each other, as well as E and F, and so on. Therefore, the clustering of these networks’ nodes (people) is high. If all friendships were similar to this, the friendships’ network would be regular. In such a network, if, for example, individual G has an exciting gossip, it takes nine steps to reach, say, individual X. These nine steps are the following: G first informs I, which would transmit the story to K, then M, O, Q, S, U, V, X.

Of course, some exceptions to the regular network of friendships may substantially impact information spread. For example, according to the rule described above, individual K may be a friend of I, J, and L. Nevertheless, in this new network version, the fourth K’s friend is Z, not M. With this exception, the network is no longer a regular one. In this case, a gossip would take just four steps to progress from G to X (G => I => K => Z => X). With a few more changes in other individuals, such as the one we introduced in K, the network becomes a “small-world” network.

In small-world networks, the clustering of nodes (people) is high, but the path of friendships between any human being is short. Rumors may spread in regular networks, but the speed would be much higher in small-world networks [28].

**Figure 2:**
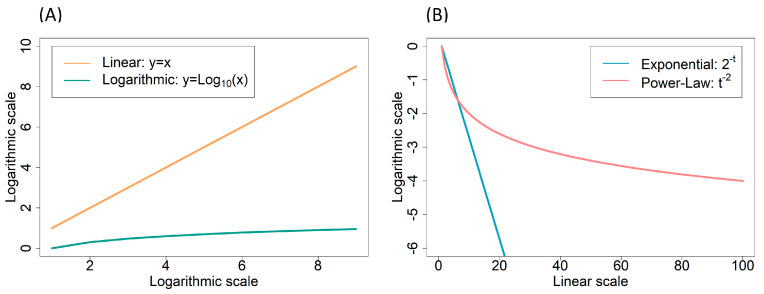
(**A**): Linear and logarithmic functions. Suppose that x increases from 1 to 10^9^, close to the world population size. Then, y increases from 1 to 10^9^ in the case of the y=x linear function, whereas the $y=Log_{10}\left(x\right)$ function increases from zero to nine only. Note that both axes are at the logarithmic scale (base 10). (**B**): Exponential and power-law decay. The exponential function 2^−t^ decreases much faster than the power-law function t^−2^. Note that the vertical axis is at the logarithmic scale (base 10) but not the horizontal axis.

What makes small-world networks so relevant to epidemiology? As mentioned above, if people organize themselves in small-world networks, the spread of information is fast because, although most people do not have direct contact with each other, most can be reached in a few steps. Suppose physical proximity or even contact is involved in these networks. In that case, microorganisms may quickly spread because the typical distance between two randomly chosen people (the network nodes) grows proportionally to its logarithm [28] instead of the number of people in the network as in regular networks. This difference is relevant because the logarithm function grows much slower than a linear function (Figure 2A). For example, when a given variable X increases from one to a billion (i.e., from 1 to 10^9^), Log_10_X goes from zero to nine only. Therefore, in small-world networks, the path between two random persons is low, even if the network contains millions or billions of people.

Of course, people spontaneously build other contact networks. For example, people in our working place are not necessarily our friends, but we contact them daily. These networks, which usually involve physical proximity or sharing a working environment, may be relevant concerning the microorganisms’ evolution or spread. For example, previous studies have shown that the shorter path lengths in small-world networks increase the effectiveness of natural selection while maintaining the fittest clones in bacterial populations because the probability of encounters between individuals is higher than in regular networks [29,30].

Liljeros et al. studied the web of human sexual connections among 2810 adult Swedish people and found that those connections defined a scale-free network [31,32]. In scale-free networks, the distribution of links in each node follows a power law [33]. In the case of the network of sexual contacts, *p*(*k*) describes the proportion of people with *k* sexual contacts in the previous year, $p\left(k\right)\approx c\cdot k^{-\alpha }$, where c and *α* are positive parameters. Therefore, all people in that Swedish database had at least one sexual partner in the previous year, but a lower fraction had two partners; an even lower fraction, some people had three sexual partners, and so on. Power-law distributions characteristically decreased slowly, so some individuals had 20 partners in the previous year. These few individuals are “hubs” of this network, which is relevant, for example, for sexually transmitted diseases [32]. In this study, the exponent α is slightly above three. The exponent *α* for women is higher than that for men, which means that the proportion of women with *k* sexual partners is lower than that of men; however, the statistical error of these estimations is higher than their difference [31,32,34]. The same authors have also shown that the cumulative distribution *P*(*k*) similarly follows a decreasing power-law distribution, $P\left(\geq k\right)\approx c\cdot k^{-\rho }$, where ρ=α−1. The word “cumulative” in the previous sentence means that $P\left(\geq k\right)$ quantifies the fraction of people with *k* or more contacts [31].

#### 2.2.2. Power-Laws and Scale-Free Networks

Power-laws are mathematical functions such as *x^q^*, where *x* is the independent variable and *q* is a negative or positive constant. They are common in physical laws; for example, the gravitational force between two bodies decreases with distance *d* according to a power law proportional to d^−2^. Similarly, the electric force between two charged bodies falls with d^−2^.

In microbiology, we also find power laws. For example, the mutation rate per replication per nucleotide of DNA-based microorganisms decreases with the genome size according to $\mu \approx c\cdot G^{-\beta }$. Because β≈−1, this equation means that the mutation rate per replication per nucleotide is inversely proportional to the genome size (μ·G≈c) so that the mutation rate per replication per genome is a constant $\left(c\approx 0.003\right)$: Drake’s rule [35,36,37]. Another example concerns the death rate of persister bacterial populations in the presence of a bactericidal antibiotic. Some authors have argued that the death rate of persister populations of some strains follows a power law with an exponent close to −2 [38,39]. A power-law decay is slower than an exponential decay, which means that bacterial cells under antibiotic exposure decay according to a power law can persist alive for longer, sometimes causing health problems and persistent food contamination (Figure 2B).

Concerning the spread of microorganisms through human-contact networks (i.e., with people as nodes), it is relevant to know if the networks are scale-free, that is, if the proportion of people with a *k* connection follows a power-law distribution. That would mean that a non-negligible proportion of people have many connections, as we have seen with the web of sexual contacts [31]. However, there are more examples of scale-free networks relevant to epidemiology.

In 2006, Brockmann et al. found something striking concerning the dispersal of banknotes in the USA. They studied banknote dispersion as a proxy of people traveling. As intuitively expected, most banknotes travel less than 10 km in four days; also, according to our intuition, the number of banknotes detected further away decreases when the distance increases. Gaussian or Exponential distributions would mostly predict that none or very few banknotes travel more than a few hundred kilometers. However, contrary to common intuition, many banknotes travel thousands of kilometers in those four days. Banknote traveling follows a decreasing power law [40]. The transactions of these banknotes may be twofold in their relevance to epidemiological studies: (i) banknotes move between physically close people, enabling cross-contagion with microorganisms; (ii) banknotes may carry microorganisms, so a person may contaminate another one without being physically close.

Tracking the position of 100,000 mobile phones for six months provides a similar distribution [41]. Most mobile phones only travel a few kilometers, and the proportion of phones traveling decays when the distance increases. A non-negligible number of cell phones traveled hundreds of kilometers. As we have seen for banknotes, the overall traveling of mobile phones follows a decreasing power law. These long-distance traveling people (measured through their banknotes and mobile phones) may constitute relevant microorganism spreaders.

As we have seen, networks where the proportion of people with *k* connections decreases according to $p\left(k\right)\approx k^{-\alpha }$, where *α* is a positive fixed number are epidemiologically relevant. However, concerning the diseases’ spread, some are even more relevant such as those power laws where the parameter *α* is between two and three. We have seen above that a suitable parameter commonly discussed in epidemiological studies is that of *R_0_*, which informs us how many people a single infected person will transmit the infection to on average in a fully susceptible population. Strikingly, there is no such threshold in networks whose connections between people follow a decreasing power-law distribution with 2<α<3. Therefore, an epidemic spread may occur even with low rates of disease transmission between the hosts [42,43]. Networks with α<3 have very high standard deviations in terms of the number of connections to each node. Therefore, in the context of the equation $R_{0}=\beta \cdot c\cdot d$, the number of contacts between infected and susceptible individuals per time unit, *c*, may also be extremely high.

If the human population network structure (small word, sometimes following a power-law distribution) somewhat facilitates microorganism spread, why do novel pathogens not almost instantly infect humans worldwide? In the case of scale-free networks, the α parameter mentioned above is sometimes lower than two or above three. For example, we have seen above that, in the case of sexual partners in the previous year, that α is slightly higher than three [31,32]. With the α parameter outside that interval, the network is still a small world, but there is an epidemic transition value [34]. Moreover, real-life scale-free networks are finite (i.e., have a limited number of people), which implies that, even if the α parameter falls between two and three, there is a non-null epidemic threshold.

Moreover, humans do not become infectious immediately after contagion, which may take a few days, depending on the disease. Furthermore, people are not permanently in contact with each other, particularly if they feel ill. Additionally, people, medical doctors, and the government commonly implement measures to halt disease spread. Even so, we have seen that with, for example, the COVID-19 pandemic, and despite arduous efforts employed by the governments of several countries, two and a half months (between December 2019 and the first days of March 2020) were sufficient to spread the SARS-CoV-2 virus to most countries worldwide. Governments employed compulsory confinements and other demanding measures because COVID-19 would kill many people and cause morbidity to many others [44].

### 2.3. Selection and Weak Counter-Selection of Virulence and Resistance Genes

Proteins encoded by virulence genes—e.g., toxins and cell surface proteins that enable bacterial attachment to host tissues, among others—can help bacteria colonize hosts [45]. Therefore, newly acquired virulence genes may not affect fitness or confer immediate advantages to bacteria, while recently acquired resistance determinants often impose a fitness cost on the bacterial cell. Therefore, these gene types may have different effects on their new hosts when newly acquired. However, the claim that resistant determinants are costly is somewhat complex.

The fitness costs of drug resistance determinants may evolve towards lower values or even become zero. For example, compensatory mutations often arise and diminish or even eliminate the deleterious effects of resistance mutations [46,47,48,49,50,51,52,53,54,55,56]. Moreover, resistance determinants (mutations or plasmids) can decrease the fitness cost of other resistant determinants, e.g., through epistatic interactions [57,58,59]. Furthermore, plasmid–plasmid interactions may facilitate plasmid transfer [60,61,62], sometimes compensating for plasmid costs [63,64].

Sometimes compensatory mutations arise in the resistance-encoding mobile genetic element, not the bacterial chromosome. In this case, when moving into another bacterial host, this genetic element carries both the resistance gene and the compensatory mutations, therefore imposing no cost on its new host [52,65].

A recent study involved 9275 patients during hospital stays over two years at the Ramon y Cajal University Hospital (Madrid, Spain). Alonso-del Valle et al. measured the cost of *pOXA-48_K8*: a naturally isolated plasmid. This plasmid was the most successful *pOXA-48*-like plasmid in an extensive collection of extended-spectrum ß-lactamase- and carbapenemase-producing enterobacteria isolated from the gut of 105 out of those 9275 patients (1.13%). The authors introduced this plasmid in 25 isolates of *Klebsiella pneumoniae* and 25 of *Escherichia coli* naïve to the *pOXA-48_K8* plasmid to determine the distribution of fitness effects on the plasmid in those 50 strains. These strains were isolated from patients coinciding on the hospital ward with others colonized with bacteria harboring *pOXA-48*-like plasmids [66].

As expected, the plasmid imposed a fitness cost to bacterial cells: a mean cost of 2.9%. Although small, this effect is statistically significant. However, individual values varied considerably. Most fitness effects were null, and the authors only observed a fitness cost in 14 strains (28%). Interestingly, the plasmid conferred a fitness advantage to the host in seven strains (14%) [66]. It is crucial to note that, even if the plasmid decreases bacterial fitness in 28% of the strains, the plasmid can succeed and “amplify” in the other 58% of the strains where the plasmid is neutral or among the 14% where the plasmid confers a fitness benefit [7,27,67].

Alonso-del Valle et al. measured the fitness of plasmid-bearing strains through competition assays. This method is appropriate because it mimics the real competition taking place between plasmid-bearing and plasmid-free cells in the gut microbiota. These competition assays were performed in agitated liquid media to avoid plasmid transfer into the competitor [66].

All the accumulated knowledge about the cost of resistant determinants points in the same direction: even if plasmid-encoded resistance determinants impose a fitness cost to naïve recipient cells, soon the cost diminishes or disappears. Therefore, resistant cells may avoid being outcompeted by sensitive cells after a critical adaptation period to a resistance-encoding gene.

One must remember that human tissues and the gut are structured media with minimal or no agitation. In such environments, plasmids can transfer to neighboring naïve cells. Possibly, the plasmid imposes a fitness cost to their neighbors, leaving resources to the donor cells. Therefore, even if plasmids are costly in the donor cells, these may succeed by imposing fitness costs on adjacent competitors, acting as harmful agents [68]. This harming behavior is advantageous to donor cells for the following reasons [68]: (i) donor cells have already adapted to the plasmid presence due, for example, to compensatory mutations [56]; (ii) the plasmid transfers to recipient cells (transconjugant cells); (iii) these cells are not adapted to the plasmid presence because they never harbored it; (iv) these non-adapted transconjugant cells replicate slower and use fewer resources than before the plasmid arrival; (v) donor cells may uptake the unused nutrients and replicate.

Furthermore, it is interesting to note that, just because they are transferable (and not due to the gain of additional genes), conjugative plasmids may have an adaptive value to their hosts, not only as harmful agents, as explained above [68], but also as promoters of bacterial biofilms, which confer protection against antimicrobials [63,69,70].

Therefore, epidemiological or evolutionary studies of antibiotic resistance must assume that resistance genes are widespread worldwide. For over eight decades, tons of antibiotics have been used and thrown into the environment [71]. The prolonged use of these drugs has promoted the clonal expansion of resistant cells and the worldwide spread of resistant clones and putative mobile genetic elements carrying resistance genes. Not surprisingly, nowadays, many metagenomes, including human and environmental ones, contain resistance genes; sometimes, this includes a diverse set of those genes as well as virulence genes [72].

### 2.4. The Diversity of Virulence and Resistance Genes across Microbiomes

We have argued above that non-housekeeping genes are somewhat free to spread within and between microbiomes (namely between people). Some people may use antibiotics, which select virulence genes that are present in resistant cells and resistance genes but counter-select other genes, including those that confer resistance to unrelated antibiotics and virulence genes encoded in bacterial cells susceptible to the used drug.

The use of antibiotics by sick people may select antibiotic-resistant pathogens, which, by definition, encode virulence genes. Perhaps this is why several studies have shown antibiotic and virulence genes co-occurrence in bacterial genomes [73,74,75]. In general, one could predict that the continuing use of tons of antibiotics through the decades would co-select virulence and resistance genes. However, it is also manifest that the administration of antibiotics hits several other bacteria, namely commensal and mutualistic bacteria, in the human microbiome [76,77,78]. Moreover, the use of antibiotics as growth promoters in livestock and agriculture or with prophylactic purposes may select resistant cells independently on whether or not they encode virulence genes [79].

Paradoxically, however, the diversity of resistance genes correlates positively with the diversity of virulence genes across human and environmental microbiomes [72]: microbiomes with a high diversity of one class of genes tend to have a high diversity of the other class. However, Darmancier et al. studied 16,632 bacterial genomes and concluded that no such positive correlation exists at the level of genomes, neither across chromosomes nor across plasmids [80]. The results of these two studies (references [72,80]) are compatible if, and only if, metagenomes with a high diversity of both gene types have those genes mostly located in different genomes. Why should there be a positive correlation at the level of metagenomes but not at the genome level?

In the previous two sections, we have also argued that virulence and resistance genes have conditions to remain in metagenomes for a long time. Each person receives diverse genes of both types during this period, accumulating them in their microbiomes, although not necessarily in the same bacterial cells. Meanwhile, pathogenic bacteria circulate through the human population. Some of those ill people take an antibiotic, which selects cells resistant to it but kills susceptible cells, including those resistant to other drugs. Therefore, a consequence of taking the antibiotic is a decrease in the diversity of drug-resistance genes [81]. Moreover, virulence genes are present in dead cells, decreasing their diversity. This process implies that people who took antibiotics recently are those with the lowest diversity of both resistant and virulence cells [82]. On the other hand, those that took antibiotics a long time ago have the highest diversity of both gene types [82]. The overall result is a positive correlation between the diversities of both gene types across human microbiomes [72,82].

What if there is a misuse (and overuse) of antibiotics, where some people use them even if they are not infected by a bacterial pathogen (or are randomly in contact with antibiotics from environmental contamination)? The process is very similar to the one described above, and the overall result is the same: a positive correlation of both genes’ diversities. These are the predictions if the probability that a metagenome loses resistance determinants is lower than the transmission probability between people [82]. We have seen above how unlikely it is that metagenomes lose resistance determinants, so the transmission probability between people must prevail or even the contamination of people from the environment or, for example, non-cooked vegetables [83].

A computer model enabled the analysis and corroboration of the above predictions [82]. The model simulated the transfer of pathogenic bacteria and one hundred categories of resistance and virulence genes. Simulations ran with different network types (regular, small-world, and random), and the results were always similar (the only difference being that the simulations reached stable results much sooner in small-world and random networks than in the regular network as expected, and according to explanations given above). Control simulations included changing the number of interacting people, the number of virulence and resistance genes, the relative probabilities of losing genes and acquiring them through contacts, or even changing the initial number of people already containing virulence and resistant genes, providing similar results [82].

Until this point, we have shown why the arguments (i) to (iii) presented in the Introduction section are wrong. About the fourth one, our discussion above does not predict a co-location of virulence and resistance genes in bacterial genomes; moreover, the computer model did not need to postulate their co-location to explain the positive correlation between the resistance and virulence genes’ diversities in microbiomes observed over human microbiomes. However, one must ask whether the last eighty years of intensive antibiotic use have put virulence and resistance genes together. As mentioned above, there is no correlation between resistance and virulence genes’ diversities throughout genomes [80]. Nevertheless, there are indirect signs of the selection of antibiotic-resistant pathogens, given that some categories of resistance and virulence genes preferentially occur in the same genome [80]. For example, Darmancier et al. observed the co-occurrence between type VII secretion systems and fusidic acid resistance genes [80]: fusidic acid is used, e.g., to treat methicillin-resistant *S. aureus* infections, and is also active against tuberculosis [84,85].

## 3. Conclusions

We have known for decades that bacteria share some core genes (a common gene pool), so health professionals and microbiologists must consider the impact of commensal bacteria on pathogenesis and public health studies [5,7,9,10,86]. Moreover, non-pathogenic bacteria may contain virulence, drug resistance, and other non-housekeeping genes that are capable of increasing the pathogens’ success during infection [6,11]. Furthermore, non-pathogenic cells that do not carry those genes may help pathogenic cells to receive those genes by amplifying their presence among microbiomes [7,27]. Finally, physical contact networks involving humans (see the above Section 2.1 and Section 2.2) facilitate the pathogens’ spread through our species. However, the vast majority of bacteria in human microbiomes, the non-pathogenic ones, must play critical roles in spreading virulence and resistance genes (Section 2.3 and Section 2.4).

## Data Availability

Not applicable.
